# Establishing a Receptor Binding Assay for Ciguatoxins: Challenges, Assay Performance and Application

**DOI:** 10.3390/toxins16010060

**Published:** 2024-01-22

**Authors:** Lisbet Díaz-Asencio, Donaida Chamero-Lago, Gabriel L. Rojas-Abrahantes, Carlos M. Alonso-Hernández, Marie-Yasmine Dechraoui Bottein

**Affiliations:** 1 Centro de Estudios Ambientales de Cienfuegos (CEAC), Carretera a Castillo de Jagua Km 1 ½ Ciudad Nuclear, Cienfuegos 59350, Cuba; donaida@ceac.cu (D.C.-L.); gabriel@ceac.cu (G.L.R.-A.); 2 Marine Environment Laboratories, Department of Nuclear Science and Application, International Atomic Energy Agency, 4 Quai Antoine 1er, 98000 Monaco, Monaco; c.m.alonso-hernandez@iaea.org; 3 University of Cote d’Azur, CNRS, ECOSEAS, 28 Avenue Valrose, 06103 Nice, France

**Keywords:** ciguatera, detection method, SIDS, technology transfer, radioligand, quality control

## Abstract

Ciguatera, a global issue, lacks adequate capacity for ciguatoxin analysis in most affected countries. The Caribbean region, known for its endemic ciguatera and being home to a majority of the global small island developing states, particularly needs established methods for ciguatoxin detection in seafood and the environment. The radioligand receptor binding assay (r-RBA) is among the in vitro bioassays currently used for ciguatoxin analysis; however, similarly to the other chemical-based or bioassays that have been developed, it faces challenges due to limited standards and interlaboratory comparisons. This work presents a single laboratory validation of an r-RBA developed in a Cuban laboratory while characterizing the performance of the liquid scintillation counter instrument as a key external parameter. The results obtained show the assay is precise, accurate and robust, confirming its potential as a routine screening method for the detection and quantification of ciguatoxins. The new method will aid in identifying high-risk ciguatoxic fish in Cuba and the Caribbean region, supporting monitoring and scientific management of ciguatera and the development of early warning systems to enhance food safety and food security, and promote fair trade fisheries.

## 1. Introduction

Radioligand receptor binding assays (r-RBA) coupled to scintillation technology have been developed and utilized since the 1990s to detect various classes of marine algal toxins [1,2,3]. These pharmacological assays rely on the binding affinity of toxins to specific biological receptors, allowing the measurement of their combined toxic potency in complex samples containing multiple related toxin congeners [4,5,6]. The introduction of a high-throughput microplate format later provided a convenient way for monitoring purposes and potential regulatory application, particularly when analyzing large numbers of samples within a short timeframe [7,8].

**Figure 1:**
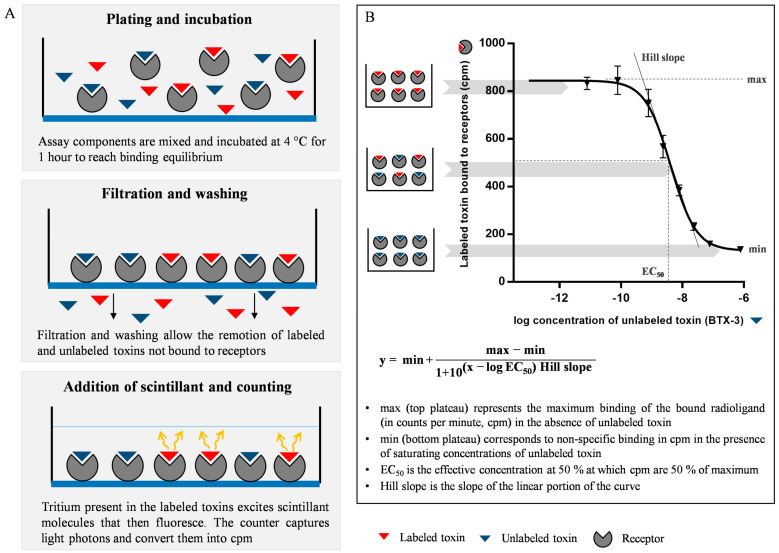
Diagram showing the experimental protocol and data acquisition during a receptor binding assay. (**A**) Schematic representation of the main experimental steps within one of the 96 wells of the plate. (**B**) Illustration of a sigmoidal dose-response curve for the brevetoxin-3 (BTX-3). Plate wells on the left depict three scenarios (suitable for purified toxin, reference material, or toxin-contaminated sample extract) ranging from absence or low concentrations of unlabeled toxins to the presence of saturating concentrations of unlabeled toxins. Labeled toxin bound to receptors in the y-axis are expressed in counts per minutes (cpm).

The principle of the r-RBA relies on the competition between an unlabeled toxin and a tritiated toxin for a finite number of available receptor sites provided by a brain membrane preparation. The binding of the radioligand to the receptor sites is proportionally reduced in the presence of increasing concentrations of the unlabeled toxin, and it is evaluated by the measurement of tritium, a low-energy beta emitter (18.6 keV), using the liquid scintillation counting method. A sigmoidal competition curve can then be constructed by measuring the concentration of the radioligand receptor complex across a range of concentrations of toxin standard. Dose–response curve fitting is performed using a four-parameter logistic fit with a variable slope or Hill equation [9], from which the amount of toxin in an unknown sample is calculated. Figure 1 shows a schematic representation of this assay.

The microplate r-RBA developed for paralytic shellfish toxins (PST), algal-derived neurotoxins targeting voltage-gated sodium channels, has been, since 2012, an AOAC Official Method of Analysis [10] and is recognized as an approved method for PST monitoring in shellfish by the Food and Drug Administration in the USA. A receptor binding assay was also developed for the brevetoxin (BTX) and ciguatoxin (CTX) groups of algal toxins [3,11], neurotoxins also targeting the same protein as the PSTs.

The r-RBA for CTX analysis, though assessed through single-lab validation [12], remains to be validated through interlaboratory studies. In general, the validation of CTX analytical methods, including the r-RBA and other chemical-based or bioassays that have been developed to detect and quantify CTX in food matrices [13,14,15,16], have been hampered mostly by the scarcity of standards and reference materials (in particular for CTX from the Atlantic and Caribbean region), and limited interlaboratory comparison [17,18]. As a result, no legally enforceable guidance has yet been provided in terms of analytical methods or permissible levels.

CTXs are responsible for ciguatera poisoning, a foodborne intoxication nowadays considered the most common non-bacterial fish intoxication. It is caused by the consumption of fish or invertebrates that have accumulated CTX via food web transfer [17]. Globally, there is a limited number of countries with the capacity to monitor CTX. Some EU countries are screening high-risk fish species for CTX contamination from areas prone to ciguatera [19] as a management option. However, small island developing states (SIDS) in the Caribbean, which are directly and the most impacted by ciguatera, lack capacity for specific CTX analysis. In these ciguatera endemic countries, management strategies mainly involve banning of high-risk fish species, a measure that is generally applied in a systematic manner even though the geographic distribution of risky areas within endemic areas and the toxicity within species varies significantly. As a result, fish that could safely be consumed are removed from the market, thereby reducing access to high quality food and hence adversely affecting food security, nutrition and livelihood of local communities.

The establishment of routine monitoring for ciguatoxin in seafood and the environment has the potential to significantly enhance the sustainable use of marine resources through better characterization of ciguatera risk and the identification of safe fishing areas, or the revision of existing risky species lists. In that objective, establishing validation processes and improving the robustness of biotoxin monitoring tools are essential steps. This study describes a single-lab validation of the r-RBA in a Cuban laboratory, including the assessment of external key parameters such as the beta counter.

In this work, a more accessible microplate beta counter option is used, equipped with liquid scintillation technology (Plate CHAMELEON V Multilabel Counter, Hidex, Turku, Finland). The choice was based on its lower cost and reduced complexity compared to the microplate beta counters commonly used in r-RBA applications (namely the MicroBeta and TopCount, PerkinElmer, Waltham, MA, USA). The methodology used in previous publications [12,20] was first adapted to suit the specific characteristics of this instrument. Subsequently, the performance of the assay was characterized by analyzing the critical parameters of the calibration curve. Twenty-four samples of high-risk fish species captured in a ciguatera hot spot in Cuba were extracted and analyzed using the newly established RBA.

## 2. Results

### 2.1. Optimization of Counting Measurements on CHAMELEON V Scintillation Counter

**Figure 2:**
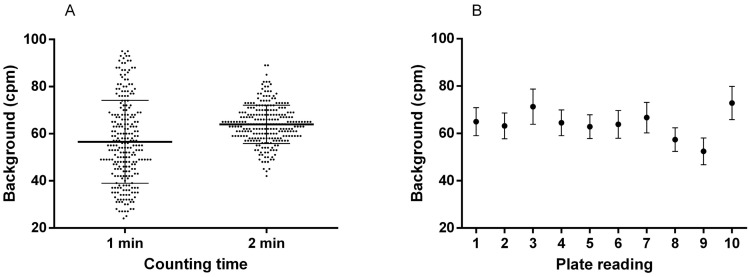
Assessment of instrument background variability. (**A**) Pooled instrument background cpm values over 25 wells and 10 plate readings for 1 and 2 min counts. Dark horizontal lines and whiskers represent global mean and standard deviation over 250 individual values. (**B**) Control chart showing averaged instrument background (two-minute counts) over 10 plate readings. Whiskers represent SD over 25 individual measurements.

The counting background variability was first assessed counting twenty-five random wells of a 96-well filter microplate (without the addition of scintillant) for one (as previously recommended) and for two minutes because of the high background announced by the supplier. There were significant differences in cpm mean values between the two counting times (unpaired *t* test, *p* < 0.0001). A very high variability was observed when counting for one minute with extreme values of 24 and 95 cpm (Figure 2A).

Averaged instrument background values ranged between 52.4 and 72.8 cpm when counting for two minutes over 10 plate readings, with a global mean of 63.9 ± 5.9 cpm (Figure 2B). Relative standard deviation within each plate reading ranged between 8.01 and 10.7% with a mean value of 9.3%. Counting for two minutes improved the repeatability of the measures and was thus chosen for the development of the RBA.

Three different scintillant cocktails, MaxiLight, Optiphase and AquaLight, were compared while assessing the counting efficiency of the instrument using 96-well black/white Isoplates and 100 μL of cocktail volume. There were significant differences in cpm values among the three cocktails tested (Kruskal–Wallis test; *p* = 0.0002). Total counts increased from about 1000 to 4000 cpm when using Optiphase versus MaxiLight. Using MaxiLight, the counting efficiency on 96-well black/white Isoplate reached 30% under the conditions tested. Consequently, MaxiLight was chosen as the scintillation cocktail to use in the RBA protocol.

There was no significant difference in cpm values between the addition of 50 and 30 µL of MaxiLight cocktail in 96-well filter microplate (Mann–Whitney test; *p* = 0.2). The volume was therefore reduced from that of the previously published protocols, i.e., from 50 µL to 30 µL, to reduce the volume of liquid radioactive waste.

### 2.2. Receptor Binding Assay Performance

**Figure 3:**
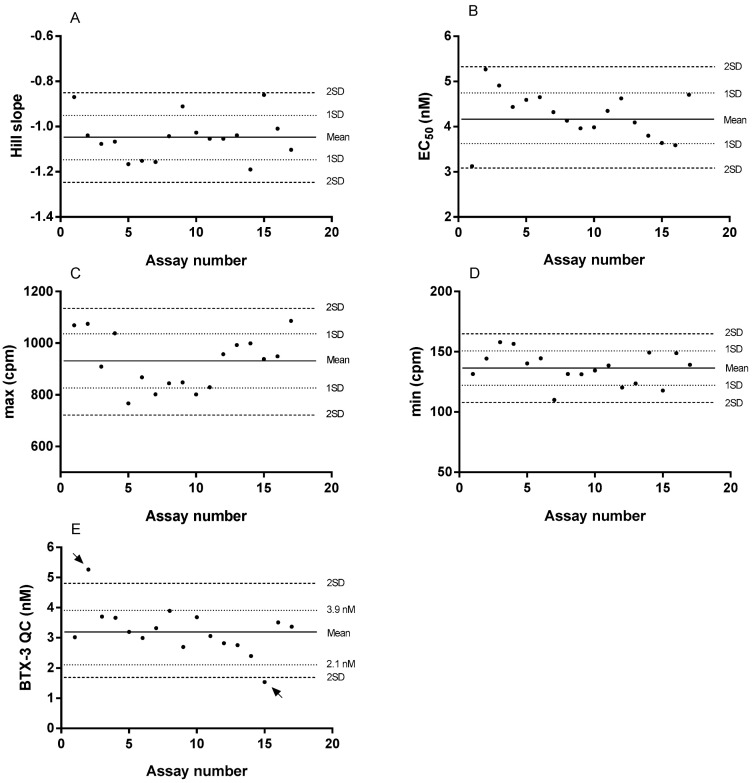
Control charts of the curve-derived parameters Hill slope (**A**), EC_50_ (**B**), max (**C**), min (**D**) and internal quality control (**E**) of the receptor binding assay. Control limits were based on the mean ± 2SD of the 17 data sets after validating the assumption of normality. Control limits for QC were also based on the 30% of 3 nM BTX-3 of the 17 data sets. The arrows at (**E**) show the outlier QC values.

The precision of the data, expressed by the relative standard deviation (RSD) among the triplicate measurements for the standards and QC data points, averaged 6.3% (±3.7, *n* = 185). Individual Hill slope and EC_50_ values calculated from 17 experiments were in the range encompassed by 2SD (i.e., no outlier was identified) and were visually illustrated using control charts (Figure 3A,B). Additionally, Hill slope and EC_50_ values complied with the quality control criteria, with requirement of 20% variability around the −1 value for the Hill slope and 30% variability around the calculated mean, for the EC_50_.

Out of the 17 QC individual values, two were outside the range encompassed by 2SD. The same two points were either below 2.1 nM or above 3.9 nM, values that state the 3% of the expected values of 3 nM (Figure 3E). Hence, these two outliers and, according to the assay acceptance rule, the corresponding curve data were removed before computation of the mean values.

The means of Hill slope and EC_50_ were −1.06 ± 0.09 (RSD = 8.4%; *n* = 15) and 4.21 ± 0.50 nM BTX-3 (RSD = 11.9%; *n* = 15), respectively. The calculated maximum and minimum binding averaged 917 ± 104 cpm (RSD = 11.3%; *n* = 15) and 137 ± 14 cpm (RSD = 10.1%; *n* = 15), respectively. QC averaged 3.2 ± 0.44 nM BTX-3 (RSD = 13.8%; *n* = 15).

The variability within sample triplicate measurements averaged 7.4% (±4.1, *n* = 126). Upper (EC_80_) and lower (EC_20_) limits were quantified for each individual experiment and used to determine the quantification range. Accordingly, sample falling below EC_80_ were reported as below the LOQ. Quantified cpm values for samples below (RBA^+^) and above (RBA^−^) individual EC_80_ expressed in cpm are presented as Supplementary Materials.

Mean upper (EC_80_) and lower (EC_20_) limits were determined to be 761 ± 84 cpm and 293 ± 26 cpm, respectively (*n* = 15 individual experiments), and the LOQ was estimated to be 1.72 ng BTX-3 equivalent g^−1^ tissue.

### 2.3. Toxicity Analysis of Fish Samples

The developed r-RBA protocol was applied to detect and quantify CTX in 24 fish captured in an area known to be at risk for ciguatera. Eighteen samples (75%) presented toxin levels higher than the LOQ of the assay and were classified as RBA^+^ (Table 1).

The concentrations of ciguatoxins in the analyzed individuals ranged from 2.8 to 8.3 ng BTX-3 equivalents g^−1^ fish. All four species collected in the study, which were identified as high risk according to the Cuban regulation [21] exhibited positive results in the RBA (RBA^+^) (Table 1). The highest toxicity value corresponded to a specimen of *Mycteroperca venenosa* (Linnaeus, 1758).

## 3. Discussion

This work presents the development and operation of the ciguatoxin-receptor binding assay in a laboratory located in Cuba. It evaluates the assay reproducibility and repeatability while also assessing its robustness with the focus of promoting the effective transfer, adoption and application of a reliable and accessible method for CTX analysis in SIDS and other lower economy countries [22] threatened by ciguatera poisoning.

The r-RBA is a specific and sensitive functional bioassay that fulfils the requirements of high-throughput and quantitative analysis. Even if the use of radioactivity could be perceived as a limitation, the routine use of the r-RBA is relevant in those laboratories where the instruments are available and where guidelines of a radiation protection program can be met. In addition, the microplate format of the r-RBA is versatile and can be used as an AOAC-validated method for paralytic shellfish toxins testing [10]. The approach taken in this study was to use a more accessible microplate beta counter option, with lower cost and reduced complexity and assess assay performance by analyzing and, when possible, comparing critical parameters with those of the commonly used beta counters [12].

Plate CHAMELEON V Scintillation Counter is not equipped with an automatic plate transporter; therefore, when counting time per well is set at two minutes, the throughput of the assay is lower in comparison to PerkinElmer counters, Microbeta and TopCount. Using Microbeta, for example, the r-RBA requires only three hours to process a full plate, and one analyst can run an estimated 32 samples per day, with up to eight samples per plate run in triplicate at two dilutions [12]. However, a total of 16 samples per day can be analyzed with the Hidex CHAMELEON V counter.

As stated in the instrument specifications, the background in the CHAMELEON V counter was less than 100 cpm, a relatively high value in comparison to the values frequently found in the Microbeta counter of less than 10 cpm. RSD within each plate reading ranged around approximately 10%, which makes background subtraction unnecessary while processing r-RBA data, taking into account that the accepted variability among replicated cpm values is 30% [10]. Some evidence suggests that the natural background in this instrument is affected by external factors such as temperature and illumination. Although this was not experimentally tested, and considering that the user manual does not give information about optimal environmental conditions for proper instrument functioning, we propose to include the assessment of background variability as a permanent control in the experimental protocol before counting an assay plate.

The minimum binding value of the sigmoidal curve obtained with the CHAMELEON V counter reflects the combination of background and non-specific binding. The averaged background value obtained in this study allows the estimation of non-specific binding at around 70 cpm. This non-specific binding value is comparable to what was found in a previous study [12], where a standard of BTX-3 was used to estimate non-specific binding using a low background Microbeta counter.

The maximum binding observed in this study, also affected by background levels, exhibited a higher variability compared to the findings reported by [12]. These authors found no differences in determining the upper limit of quantification of the r-RBA using the standard deviation associated with maximum binding (max − 10 × SD of max) and using the EC_80_. In the current study, the EC_80_ and EC_20_ were used to delimit the quantification range in each experiment. The importance of monitoring the maximum as an assay performance acceptance criterion was raised for an RBA protocol developed for saxitoxins. The authors noted [23] that variability in the maximum can pose challenges in the RBA, occasionally occurring when one or more of the lowest three standards are not within control limits.

The results presented here show the r-RBA is precise and accurate when using a 96-well microplate format with brevetoxin standard, confirming its potential as a routine screening method for the detection and quantification of ciguatoxins. The precision of standards, QC and sample data, expressed by the RSD among the triplicate measurements, were well below the accepted cut-off value 30% [10]. All individual Hill slope values were in the range encompassed by 20% of the expected theoretical value of −1, that is, between −0.8 and −1.2 [10]. The variability of EC_50_ was below the accepted cut-off value 30%, and was lower than the value obtained by [12]. The EC_50_ mean value was in the range obtained by other authors [24,25] using different RBA protocols, which evidence the robustness of the assay. The mean Hill slope, EC_50_ and QC values obtained in the study can be used as reference values for the assessment of assay performance when samples are analyzed.

The results obtained demonstrate that this modified r-RBA protocol can be used for the routine analysis of CTX in fish samples. Additionally, the use of BTX as a standard offers advantages in terms of technique sustainability, given its commercial availability and greater cost-effectiveness (300 to 400 times lower) compared to CTX standards. It is worth noting that currently the only commercially available standards for CTX are of the Pacific types (e.g., P-CTX3C, P-CTX1B), while there are no available commercial standards for Caribbean type of CTXs (C-CTX).

The conversion of analytical results from the BTX equivalent (or P-CTX3C equivalent) to C-CTX remains a challenging task due to the limited information available regarding toxicity equivalency factors for both BTX or CTX analogues. To our knowledge, only one published study by [24] has provided insights into the differences between BTX-3 and C-CTX1 affinity for voltage-gated sodium channels. This study reveals an eight-fold-higher affinity for C-CTX1 compared to the BTX-3, with reported EC_50_ values of 0.34 and 2.77 ng mL^−1^ in the RBA, respectively. Consequently, considering this information, the concentration values quantified in the present study would be eight times lower if expressed in C-CTX1 equivalents.

Ciguatoxin concentration in the analyzed fish individuals, expressed as BTX-3 equivalents, ranged from 2.8 to 8.3 ng g^−1^. It is now well established that fish in Cuba may accumulate CTX [26]; therefore, the inhibition of tritiated brevetoxin by the extracts in the r-RBA is attributed to the presence of ciguatoxin. Although the possibility of BTX presence should not be ruled out, as these toxins can also occur in marine fish [27], to our knowledge, no blooms of *Karenia brevis* or other brevetoxin-producing microalgae (such as *Chatonella* spp., *Heterosigma akashiwo* and *Fibrocapsa japonica* [28]) have been reported in the sampled area.

Applying the conversion factor mentioned above, the estimated concentration in equivalents of C-CTX1 range between 0.34 and 1.02 ng g^−1^. Although these concentration values are lower than those previously reported for this study area (1.5 to 8 ng g^−1^ in equivalents of CTX3C or C-CTX1 [26]), they are above the limit recommended for the C-CTX1 of 0.1 ng g^−1^ of fish (FDA 2011), therefore anticipating a potential threat to human health. However, actual risk and limits in seafood for Caribbean ciguatoxins still need to be determined through a health risk assessment. The implementation of effective notification systems for ciguatera outbreaks that allow access to fish samples implicated in ciguatera poisoning in this region could help in achieving this goal.

In order to improve ciguatoxin detection through r-RBA, continued optimization of this approach is still required to enhance its accuracy and reliability in the future. Potential avenues for improvement include a better characterization of the fish matrix influence, estimation of measurement uncertainty and participation in intercomparison exercises. Additionally, it is highly recommended to validate the use of BTX as a reference standard in the r-RBA to allow its unequivocal conversion into equivalents of C-CTX1, for which the availability of certified standards remains essential.

Having the r-RBA established will help provide the scientific data needed to support the list of high-risk ciguatoxic fish included in the Cuban fish regulation. The monitoring and scientific management of ciguatera in Cuba can now be considered, including the development of early warning systems to support food security and promote fair trade fisheries.

## 4. Materials and Methods

To identify potential adjustment in the protocol, the first task consisted of summarizing the technical specifications of the instrument in comparison to that stated for the commonly used microplate scintillation counters MicroBeta and TopCount. These counters are among the most common microplate scintillation counters that have been traditionally used to quantify algal toxin through r-RBA [3,10,23,29]. Providing coincidence counting due to two photomultiplier tubes (PMT) positioned above and below the sample that simultaneously detect signal, MicroBeta ensure high efficiency and extremely low background for a variety of radionuclides, including tritium [30]. TopCount counters use a single PMT and feature Time-resolved counting technology, a method that discriminates between background and true counts and results in a superior sensitivity, high signal-to-noise ratios and virtually crosstalk-free counting when used with opaque plates [31]. Background values as low as 20 counts per minute (cpm) and a high counting efficiency (more than 45%) are usually achieved with both single- and dual-PMT counters, thus making background assessment and eventual average subtraction unnecessary. The specifications of the MicroBeta and TopCount counters are provided in Table 2.

The Plate CHAMELEON V Multilabel Counter supports liquid scintillation, fluorescence and luminescence technologies, with a single PMT located immediately above the sample well [32]. However, Time-Resolved technology is not available for scintillation mode; therefore, high-performance counting on opaque plates such as the filter plates used in the RBA, is not guaranteed. As per manufactured specifications, background is relatively high with values stated as less than 100 cpm. Counting efficiency for tritium reaches a maximum between 30% and 50% depending on the plates used [32], values comparable to that announced by PerkinElmer counters MicroBeta and TopCount (Table 2).

Due to the lack of information available in the scientific literature on the Plate CHAMELEON V Multilabel Counter, the first step was to characterize counting performance based on the reported manufacturer specifications. Counting time, selection of the most suitable scintillation cocktail and the volume of the cocktail to use in the assay were sequentially tested.

### 4.1. Counting Performance Assessment

The background of the beta counter was assessed using 96-well filter microplate. Twenty-five random wells were counted for one and two minutes each over ten plate readings in ten separate days. Due the fact that this counter has only one detector, additional counting times were not assessed. For example, it would take almost five hours to read a full plate assay using three-minute counts, which would decrease the throughput of the assay by half.

The instrument counting efficiency was assessed using MaxiLight cocktail (Hidex, Turku, Finland) and black/white Isoplate. MaxiLight is a lipophilic cocktail with highest counting efficiency for organic and non-aqueous samples, and dry samples and filters. Other two available cocktails (Optiphase and AquaLight) were tested and compared as well. Optiphase (Hidex, Turku, Finland) is specifically produced for micro-volume counting, and as such, it is a commonly used cocktail in r-RBA application. Aqualight (Hidex, Turku, Finland) is a general-purpose scintillation cocktail capable of handling a broad range of solutes, combining high counting efficiency and low background. A solution of tritiated brevetoxin (^3^H-PbTx-3), similar to the one used as a working solution in the r-RBA protocol (6.45 kBq mL^−1^), was used as a tracer. An amount of 35 µL of this solution (225.8 Bq equivalent to 13,545 disintegrations per minute, dpm) and 100 µL of each cocktail were added to a 96-well black/white Isoplate in four replicates and counted for two minutes.

Two different volumes of cocktail were tested on 96-well filter microplate (MultiScreen HTS FB Filter Plate MSFBN6B50, Millipore, Burlington, MA, USA): the original protocol’s volume of 50 µL, and a reduced volume of 30 µL that adequately covered the well bottom and allowed for easy pipetting of the viscous solution using multichannel pipettor. Four replicates of 3.5 µL of the tracer solution (22.6 Bq equivalent to 1354.5 dpm) were added to the wells containing MaxiLight cocktail and counted for two minutes.

### 4.2. Receptor Binding Assay Protocol

Calibrations standards and a quality control (QC) of brevetoxin (BTX) were used in this study as per [20]. The r-RBA experimental protocol was the one described in [12] with some modifications. Analytical grade chemicals and HPLC-grade solvents were used throughout the study. A stock solution of brevetoxin 3 (known as BTX-3 or PbTx-3) provided at 1 µg µL^−1^ (American Radiolabeled Chemical Inc., St. Louis, MO, USA) was used to prepare the calibration curve standards. Working bulk solutions (ranging from 0.06 ng mL^−1^ to 6 µg mL^−1^) were prepared by serial dilution in Phosphate-Buffered Saline Tween 20 (PBST buffer, pH 7.4, Sigma Aldrich, St. Louis, MO, USA) to reach final in-assay concentrations from 0.007 to 700 ng mL^−1^. Similarly, the QC was prepared to reach a final in-assay concentration of 2.7 ng mL^−1^ (3 nM). The use of bulk reference dilutions minimizes the pipetting needed for setting up an assay routinely and improves day-to-day repeatability. They were prepared in advance and stored at 4 °C for up to 1 month. A buffer control was run with each BTX-3 standard curve as a negative control parameter that allows inter-assay comparison. The radiotracer ^3^H-PbTx-3 was provided at 20 Ci mmol^−1^, and 0.05 mCi mL^−1^ (American Radiolabeled Chemicals Inc., St. Louis, MO, USA). A working solution was prepared containing 8.75 nM of ^3^H-PbTx-3 (6.45 kBq mL^−1^) in PBST buffer with bovine serum albumin (PBST/BSA; BSA 1 g L^−1^) for a final in-well concentration of 1 nM. A 2 mL aliquot of porcine brain membrane homogenate (Sigma Aldrich, St. Louis, MO, USA) was diluted before plating in 24.5 mL of PBST/BSA to yield approximately 0.8 mg mL^−1^ protein concentration in the assay.

To perform the assay, 35 μL of PBST/BSA was first added to each well in a 96-well microtiter filter plate (MultiScreen HTS FB Filter Plate MSFBN6B50, Millipore, Burlington, MA, USA) to moisten the filter membrane. Then, 35 μL of BTX-3 standards, QC check or sample dilutions (see below) were added in triplicate to the corresponding wells. Last, 35 μL of the ^3^H-PbTx-3 working solution and 195 μL of brain membrane homogenate were added in that order to each well. The plate was then incubated for 1 h at 4 °C before filtration and rinsing twice with 200 μL ice-cold PBST on a MultiScreen HTS vacuum manifold (Millipore, Burlington, MA, USA) system. However, certain modifications were necessary compared to the original protocols due to the absence of cassette holding the plate in the new counting instrument. Following the second rinse, it was not required to remove the underdrain of the filter plate. Instead, it was directly blotted using lint-free paper towels and sealed underneath with clear sealing tape. For control purposes, 3.5 μL of the working solution containing an activity of 22.6 Bq (equivalent to 1354.5 dpm) were added to an empty well in each run. After addition of scintillation cocktail (30 μL of MaxiLight as determined in this study), the top of the plate was sealed and incubated in the dark for one hour at room temperature. Radioactivity was then counted for two minutes.

### 4.3. Data Analysis

Seventeen BTX-3 calibration curves were run. GraphPad Prism (GraphPad Software, Inc. version 6.01, La Jolla, CA, USA) was used to generate BTX-3 standard curves and to perform data analysis. The r-RBA quality control included the analysis of assay and sample measurement acceptances as proposed by [10,33] during the validation studies for saxitoxin analysis (Table 3).

Assay performance was assessed prior to curve-fitting the data, with the verification of the relative standard deviation (RSD) of toxin standard and QC triplicate data to be below 30% [10] (Table 3). The curve-derived parameters EC_50_, Hill slope, maximum binding (top plateau or max) and minimum binding (bottom plateau or min) and the QC of brevetoxin were used as assay critical control points. For each parameter, Q-Q plots were used to assess the distribution of the associated errors. Data that met assumptions of normality were then examined for outliers using the standard deviation (SD). Points that were above (Mean + 2SD) and below (Mean − 2SD) were removed. Hill slope was checked to be in a variability range of 20% around a theoretical value of −1 according to one receptor site in homologous competition experiments [10]. Additionally, EC_50_ was checked to be in a variability range of 30% [10] and the QC to be 3 nM BTX-3 (in-well concentration) ±30% of recovery (Table 3).

Following sample data inspection (RSD < 30% of triplicate cpm data), toxin concentrations were estimated against a BTX-3 standard curve. Data were transformed in concentration values using GraphPad interpolating from unknown function using the Hill equation (formula below), within the acceptable upper and lower limits EC_80_ and EC_20_, corresponding to 80 and 20% of specific binding, respectively (Figure 2), as defined in [12]. The limit of quantification (LOQ) was calculated using EC_80_ at a maximum matrix concentration of 0.6 g tissue equivalent mL^−1^ in assay as per [12].

$$y=\min+\;\frac{\max\;-\;\min}{{1+10}^{(x\;-\log\mathrm{EC}50)\;\mathrm{Hill}\;\mathrm{slope}}}$$

### 4.4. Toxicity Analyses of Fish Samples

The RBA was then tested to determine CTX concentration in fish captured in Cuba, in an area identified as prone to ciguatera [26]. Fish individuals were kindly provided by a professional fisherman. They were collected by hook and line at 20 m depth, south of Cayo Guano del Este, a reef ridge covered by corals, gorgonians and abundant macroalgae.

No endangered or protected species were involved in this study. Species controlled under Cuban regulation [21] were selected preferentially. Total length (to the nearest cm) and weight (to the nearest g) were recorded. The fish were transported on ice to the laboratory where they were morphologically identified to species level as described in the Species Identification Guide for Fishery Purposes [34]. Then, they were filleted and stored at −20 °C until ciguatoxin extraction and RBA analysis.

Crude extracts were obtained using the extraction protocol described in [12]. Briefly, tissue samples (5 g) were cooked in a water bath at 70 °C for 15 min and homogenized in acetone to extract soluble compounds. After centrifugation and drying acetone supernatants, two solvent/solvent partitions (1:1 *v*:*v*) with hexane: 90% aqueous methanol (MeOH) and dichloromethane (DCM)/60% aqueous MeOH were applied to remove lipids and separate CTXs from other concomitant toxins (e.g., maitotoxins), respectively. The resulting DCM extract was dried and resuspended in MeOH to 10 g tissue equivalent mL^−1^ and stored at −20 °C until further analysis.

## Figures and Tables

**Table 1:** List of the species analyzed in the study. RBA^+^ indicate values above limit of quantification (LOQ) of the assay.

Specie	Family	*n*	Weight (Range) kg	Total Length (Range) cm	RBA^+^	RBA^−^	BTX-3 Equiv. (Range) ng g^−1^
*Caranx latus*	Carangidae	14	2.03–6.4	55–83	11	3	<LOQ–7.6
*Mycteroperca venenosa*	Serranidae	7	2.4–5.9	57–80	4	3	<LOQ–8.3
*Seriola* sp.	Carangidae	2	10.8–na	102–na	2	0	3.1–5.1
*Sphyraena barracuda*	Sphyraenidae	1	3.8	100	1	0	4.1

RBA^+^ indicate values above limit of quantification (LOQ) of the receptor binding assay (RBA); na: not available.

**Table 2:** Comparison among different scintillation counters used for RBA applications.

	MicroBeta	TopCount	CHAMELEON V
Manufacturer	PerkinElmer	PerkinElmer	Hidex
N° of detectors	1,2, 6 or 12	2, 4, 6 or 12	1
Position of the PMTs/Coincidence counting	Upper and lower/Yes	Upper/No	Upper/No
TR-LSC using opaque plates	Yes	Yes	No
Background	<15 cpm using 1450-514 clear-bottomed Isoplates^TM^ with white frame for LSC coincidence counting	<20 cpm using 96-well filtration plate + 50 mL of MicroScint cocktail	<100 cpm
Counting efficiency for ^3^H	>45% using the Detector normalization standard plate 1450-471	Excellent counting efficiency in opaque microplates	30% using Multiscreen filter plates; 50% in black/white Isoplates
Automatic plate transporter	Yes	Yes	No
Throughput	High	High	Low

PMT: photomultiplier tube (technical specification of the PMT are not provided by the supplier); TR-LSC: Time-resolved liquid scintillation counter.

**Table 3:** Quality control points of r-RBA for CTX detection and quantification.

Assay acceptance	☑ RSD of triplicate cpm data of standards and QC < 30% ☑ Hill slope: 20% variability around a theoretical value of −1 ☑ EC_50_: 30% variability around a calculate mean value ☑ QC: 30% variability around a nominal concentration of 3 nM
Sample measurement acceptance	☑ RSD of triplicate cpm data of samples < 30% ☑ Quantification of samples: unknown fall between EC_80_ and EC_20_

## Data Availability

The original contributions presented in the study are included in the article. Further inquiries can be directed to the corresponding authors.

## Supplementary Materials

The following supporting information can be downloaded at: https://www.mdpi.com/article/10.3390/toxins16010060/s1, Figure S1: Examples of quantified counts per minute (cpm) values for different dilutions of the fish extracts (small black circles), with a distinction between values below (RBA^+^) and above (RBA^−^) the EC_80_ marked by horizontal red lines for six separate RBA experiments. The EC_20_ for each individual experiment is indicated by black solid triangles and the maximum binding values are reported (bigger black circles) with their corresponding standard deviations.
