# Supplementary material for: Establishing a Receptor Binding Assay for Ciguatoxins: Challenges, Assay Performance and Application
Source: Toxins (Basel). 2024 Jan 22;16(1):60. doi: 10.3390/toxins16010060 (PMC10818520; doi:10.3390/toxins16010060)
Supplement: Supplementary file 1 [file toxins-16-00060-s001.zip › toxins-2765028-supplementary.pdf]

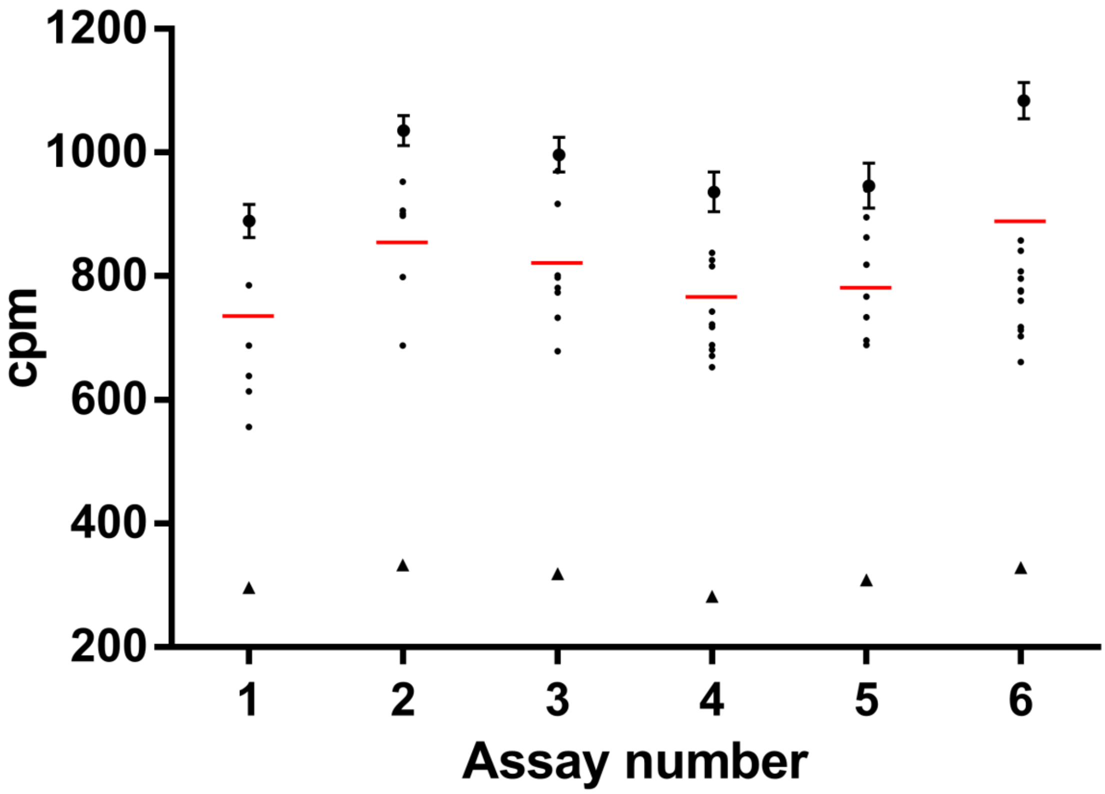

Figure S1. Examples of quantified counts per minute (cpm) values for different dilutions of the fish extracts (small black circles), with a distinction between values below (RBA^+) and above (RBA^-) the EC_{80} marked by horizontal red lines for six separate RBA experiments. The EC_{20} for each individual experiment is indicated by black solid triangles and the maximum binding values are reported (bigger black circles) with their corresponding standard deviations.
